# Differences in Fear and Negativity Levels Between Formal and Informal Health-Related Websites: Analysis of Sentiments and Emotions

**DOI:** 10.2196/55151

**Published:** 2024-08-09

**Authors:** Abigail Paradise Vit, Avi Magid

**Affiliations:** 1 Department of Information Systems, The Max Stern Yezreel Valley College Emek Yezreel Israel; 2 Management, Rambam Health Care Campus Haifa Israel

**Keywords:** emotions, sentiment, health websites, fear

## Abstract

**Background:**

Searching for web-based health-related information is frequently performed by the public and may affect public behavior regarding health decision-making. Particularly, it may result in anxiety, erroneous, and harmful self-diagnosis. Most searched health-related topics are cancer, cardiovascular diseases, and infectious diseases. A health-related web-based search may result in either formal or informal medical website, both of which may evoke feelings of fear and negativity.

**Objective:**

Our study aimed to assess whether there is a difference in fear and negativity levels between information appearing on formal and informal health-related websites.

**Methods:**

A web search was performed to retrieve the contents of websites containing symptoms of selected diseases, using selected common symptoms. Retrieved websites were classified into formal and informal websites. Fear and negativity of each content were evaluated using 3 transformer models. A fourth transformer model was fine-tuned using an existing emotion data set obtained from a web-based health community. For formal and informal websites, fear and negativity levels were aggregated. *t* tests were conducted to evaluate the differences in fear and negativity levels between formal and informal websites.

**Results:**

In this study, unique websites (N=1448) were collected, of which 534 were considered formal and 914 were considered informal. There were 1820 result pages from formal websites and 1494 result pages from informal websites. According to our findings, fear levels were statistically higher (*t*_2753_=3.331; *P*<.001) on formal websites (mean 0.388, SD 0.177) than on informal websites (mean 0.366, SD 0.168). The results also show that the level of negativity was statistically higher (*t*_2753_=2.726; *P*=.006) on formal websites (mean 0.657, SD 0.211) than on informal websites (mean 0.636, SD 0.201).

**Conclusions:**

Positive texts may increase the credibility of formal health websites and increase their usage by the general public and the public’s compliance to the recommendations. Increasing the usage of natural language processing tools before publishing health-related information to achieve a more positive and less stressful text to be disseminated to the public is recommended.

## Introduction

### Background

A wide range of medical information is available on the web and can be used by individuals who are not health care professionals to gain a better understanding of health and illness, as well as to provide an explanation for their symptoms [1,2]. However, it should be noted that health-related search information may have a significant impact on people’s decisions and concerns, including the ability to diagnose and assess their own health care needs, deciding whether or not to consult with a physician for assistance with diagnosis and treatment, and their approach to maintaining their own and their families’ health [3].

People can experience anxiety when reading information on the internet [4-6]. Exposing people without medical knowledge to complex medical terminology may put them at risk of self-diagnosis and self-treatment that could be harmful to them [7]. According to White and Horvitz [1], web search engines can escalate health concerns and various factors contribute to this escalation, including the amount and distribution of health-related content viewed by users, the presence of escalatory terminology on the pages visited, and a user’s propensity to escalate concerns in order to find more plausible explanations for their ailments. A condition known as “cyberchondria” is the creation of unfounded concerns about common symptoms based upon web-based searches and other sources of information [1].

People search health-related information on various topics. Previous studies showed that the most searched health-related topics were the cancer diseases, cardiovascular diseases, and infectious diseases [8]. Web search of health-related topics may lead to formal websites, originating from an official health organization such as the World Health Organization, and to informal websites, originating from unofficial health-related websites, including blogs of people without appropriate medical training [9]. Both formal and informal health-related websites may contain information that may increase the reader’s fear while reading the information [10]. However, whether there is a difference in fear and negativity levels between health-related information that appears in formal health-related websites and health-related information that appears in informal websites remains questionable.

Sentiment and emotion analysis are natural language processing (NLP) techniques for the identification of sentiment and emotion from speech or voice data, images, or text data [11-13]. In sentiment analysis, the overall polarity of a text is identified—whether it is positive, negative, or neutral. Emotion analysis involves the identification of specific emotions expressed in a text. In accordance with Ekman’s [14] model, several basic emotions can be identified, such as happiness, sadness, anger, disgust, surprise, and fear.

Different methods can be used to detect sentiment and emotions in a given text, and in recent years transformer models have become increasingly popular, especially in NLP. Transformer model is a deep neural network that learns context and meaning using self-attention [15]. Text classification using the transformer model achieved similar accuracy to human annotations. In recent years, several pretrained language models have been introduced, including BERT [15], XLNet [16], RoBERTa [17], DistilBERT [18], and ALBERT [19]. Our study aimed to assess the association between the type of health-related website (formal or informal) and 2 outcome variables: the level of fear and the level of negativity by using transformer for sentiment and emotion detection.

### Related Work

The analysis of emotions and sentiments in health information has become increasingly popular in recent years, particularly with the rise of social media as platforms for sharing health-related information [20]. This section provides an overview of studies that investigated the analysis of emotions and sentiments in health-related information.

### Sentiment Analysis in Web-Based Health Information

Studies have examined sentiments of patients with cancer in web-based forums [21,22]. Cabling et al [22] analyzed the feelings and opinions of web-based breast cancer support group users regarding tamoxifen, hormone-based therapy used to treat breast cancer. Results showed that the most active users were significantly more positive than the least active users, who were more negative. Users with higher cancer stages were less likely to post, focusing on side effects and associated anxiety and sadness. In contrast, users with lower cancer stages were more likely to post, remain active, and encourage social support. Carrillo-de-Albornoz et al [23] analyzed more than 3500 posts on web-based health forums concerning breast cancer, Crohn disease, and various allergies. They trained different machine learning algorithms to automatically classify patient-authored content into positive, negative, and neutral categories. Using word embeddings, they predicted the polarity of patient-authored content with an accuracy of 0.7, which indicates the times that the algorithms correctly classified the sentiment of the posts, outperforming more traditional methods.

In addition, other studies have examined sentiment analysis of patient responses online [24-27]. Greaves et al [24], for instance, used machine learning to understand patients’ comments about their care in web-based comments posted about hospitals on the English National Health Service website. A sentiment analysis technique was used to categorize web-based free-text comments made by patients as positive or negative. Ali et al [25] analyzed 26 different threads on 3 medical forums and determined whether the posts were positive, negative, or neutral regarding hearing loss.

Considering the analysis of emotions in web-based health information, Khanpour and Caragea [28] proposed ConvLexLSTM to identify fine-grained emotion types in health-related web-based posts in web-based health communities. This model combines Convolutional Neural Network features with lexicon-based features, which are fed into a Long Short Term Memory network. They demonstrate that ConvLexLSTM outperforms strong baselines and prior works. Later in 2020, Sosea and Caragea [29] presented CANCEREMO, an emotion data set created from a web-based health community and annotated with 8 fine-grained emotions. Their most effective BERT model achieves an average *F*_1_-score of 71%.

### Sentiment Analysis of Web-Based Health-Related Information on Social Media

An extensive amount of health data is disseminated via social media platforms such as Twitter and Facebook [30]. Studies in this category analyze users’ sentiments regarding disease and mental health, for example, sentiments in responses to YouTube videos related to proanorexia and antianorexia [31]. Similarly, Roccetti et al [32] explore attitudes toward Crohn disease on Facebook and Twitter. Furthermore, Ricard et al [33] examine the potential of community-generated social media content for detecting depression on Instagram.

Health discourse on social media has been significantly affected by the COVID-19 pandemic [34]. Public attitudes and opinions regarding vaccination have been the subject of several studies [35-41]. Using active learning to select a large population of health care professionals’ Twitter accounts, Elyashar et al [42] analyzed health care professionals’ web-based discussions as expressed in web-based discussions published on Twitter during the COVID-19 pandemic. According to their analysis, the intensity of emotion in their discourses decreased with the pandemic waves in 2020. They revealed a decline in joy and an increase in sadness, fear, and disgust in that year’s discourses. Aduragba et al [43] propose EmoBERT, an emotion-based variant of the BERT transformer model, able to learn emotion representations during major disease outbreaks using the Twitter platform and outperform the state-of-the-art.

Christensen et al [44] analyzed 172 major news outlets across 11 countries. As a result of keyword-based frequency analysis, the percentage of vaccination-related papers in all collated English-language papers was calculated. The Vader Python module, developed by CJ Hutto and Eric Gilbert, quantified sentiment polarization. The number of negatively polarized papers increased from 6698 in 2015-2019 to 28,552 in 2020-2021 (the COVID-19 pandemic period).

In contrast to previous studies, which analyzed textual data posted by users and their posts, reviews, and comments for sentiment analysis, our objective is to gain a better understanding of the sentiments and emotions expressed on formal and informal health-related websites. By focusing primarily on the differences in negative and fear levels expressed on formal and informal health-related websites, we hope to provide a more comprehensive understanding of fear and negative sentiment that is provided to the public through formal and informal health-related websites.

## Methods

### Overview

**Figure 1:**
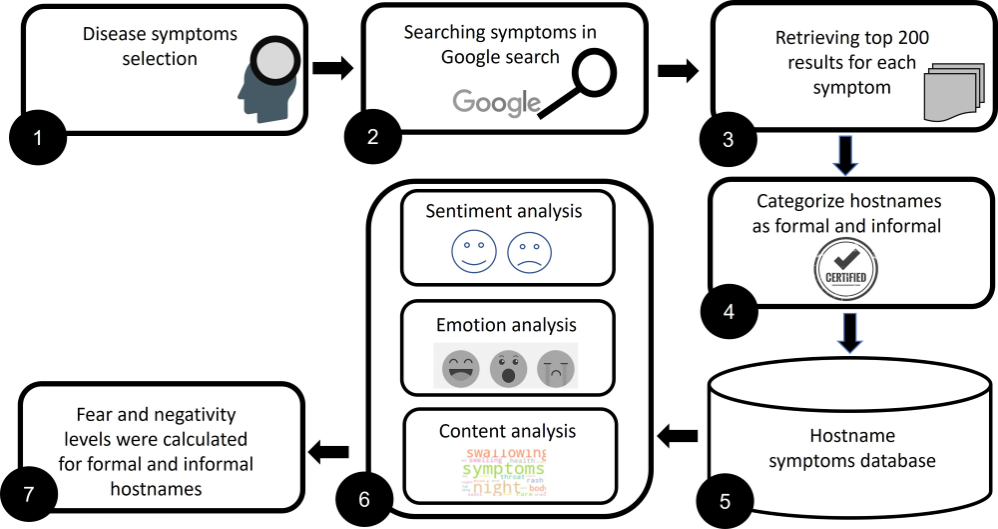
An overview of the main phases performed.

Figure 1 summarizes the seven main phases we followed to conduct the proposed study: (1) based on literature, identifying the symptoms associated with each family disease, (2) conducting Google searches for each symptom, (3) retrieving the top 200 pages returned by Google for each symptom, (4) classifying each website as formal or informal by manual labeling, (5) creating a data set of websites symptom results, (6) applying content, sentiment, and emotion analysis to the text of each website, and (7) aggregating the results to fear and negativity levels.

### Identification of Family Disease Symptoms

Common diseases were first considered, including infectious diseases, cancer, and cardiovascular diseases. Symptoms for selected diseases were identified using existing literature regarding common symptoms associated with these diseases [8-10,45,46]. The list of symptoms is shown in Table 1.

**Table 1:** Symptoms related to each disease family.

Diseases family	Related symptoms
Infectious	Fever Headache Muscle strain Cough Shortness of breath Weakness
Cancer	Bleeding Cough Chronic cough Itch Pain Back pain Rash Swelling Recurrent pneumonia Diarrhea Abdominal pain Rectal bleeding Blood in urine Difficulty or pain while swallowing solid food Vomiting Choking on food Enlarged lymph nodes Night sweats Breast tenderness Pain or burning sensation Breast warmth or redness Breast lump Breast pain or burning Weakness
Cardiovascular	Chest pain Chest pressure Chest tightness Shortness of breath

### Data Collection

We conducted a web search to retrieve websites containing symptoms of selected diseases, including infectious diseases, cancer diseases, and cardiovascular diseases. In order to search the websites, we used Google Web Search using an application programming interface provided by the RapidAPI website.

We conducted a search for each symptom and added the top 200 search results to our website’s database. Our database is a comma-separated values file containing the entire website content. We obtained the hostname for each website using the application programming interface. In this study, the term website refers to the hostname of a specific website.

We used self-written Python code (version 3.9; Python Software Foundation) to decompose each website’s content into sentences. Since long text contains multiple emotions [47], it was convenient to divide the content into sentences. We applied standard text preprocessing techniques to each content, such as removing line breaks, nonalphabetic words, stop words, and hyperlinks using natural language toolkit (NLTK, version 3.8.1).

### Ethical Considerations

The original data collection was approved by our institutional review board. This analysis received approval from The Emek Yezreel College Ethical Review Board (approval number 2024-74 YVC EMEK). The research did not involve human subjects (the research data included only websites). Therefore, informed consent was not applicable, privacy and confidentiality of the data were not applicable, and compensation details were not applicable.

### The Classification of Website as Formal or Informal

The received websites were manually categorized into 2 categories: formal websites and informal websites. A clear and consistent set of criteria based on expert domain knowledge guided the categorization process.

The criteria for formal health websites refer to those operated by an official government ministry or organization or authority, such as the ministry of health, the Centers for Disease Control and Prevention, the World Health Organization, and official websites of established health care facilities such as hospitals and academic medical centers (not including private clinics). Websites that did not meet these criteria were categorized as informal websites. Among the informal websites were blogs, news websites, entertainment websites, and so forth.

### Content Analysis

To analyze the content of each family disease, we calculated the average term frequency—inverse document frequency (TF-IDF). The TF-IDF measures the importance of words within each family disease. A term frequency (TF) measures how frequently a particular term appears in the content of a website. It is calculated by dividing the number of times a term appears in a website’s content by the number of terms found across all family disease websites. Inverse document frequency (IDF) measures the importance of a term in the entire data set of family disease. It is calculated as the logarithm of the number of websites belonging to the family disease divided by the number of websites containing the term. The TF-IDF for each term appearing on a website in family disease has been averaged across all websites of family disease to determine the average TF-IDF. The average TF-IDF measures the overall importance of terms in family disease, where higher values indicate greater significance.

### Emotion Analysis Using 3 Models

For the emotion classification task, we selected 2 transformer models from the Hugging Face Transformers repository. The models are distilled versions of state-of-the-art models of RoBERTa [17] and BERT [18], which means that they have been compressed and made smaller in size while maintaining most of the original model’s performance. These 2 models are the most downloaded among the distilled models for emotion detection. Based on diverse text classification data sets, these models have been carefully fine-tuned to capture and leverage contextual information, enabling them to effectively classify text.

1. j-hartmann/emotion-english-distilroberta-base: the model is a fine-tuned checkpoint of DistilRoBERTa-base. The model is available in the HuggingFace repository as “j-hartmann/emotion-english-distilroberta-base.” Using 6 diverse data sets, the model predicts Ekman’s 6 basic emotions, plus a neutral class: anger, disgust, joy, fear, sadness, surprise, and neutral [48].
2. bhadresh-savani/distilbert-base-uncased-emotion: we used a pretrained model for emotion detection. It is available on the HuggingFace repository under the name “bhadresh-savani/distilbert-base-uncased-emotion.” This model [18] is a distilled version of the BERT base model, fine-tuned on the emotion data set with an accuracy of 93.8% [49] of English Twitter messages with 8 basic emotions: anger, anticipation, disgust, fear, joy, sadness, surprise, and trust.

Several state-of-the-art transformer models such as RoBERTa [17], Distilbert [18], and ALBERT [19] were trained on an emotion data set of a web-based health community, annotated with 8 fine-grained emotions [29]. This data set allows us to select the best model based on accuracy and *F*_1_-scores that fit most commonly for web-based health-related information and then use the model for estimating fear emotions arising from text of new health-related data. The data set contains 5389 sentences annotated with fear or unfear emotions equally. We used these labels to measure the accuracy and *F*_1_-score of each model in predicting the fear emotion and choose the model with the best performance. distillbert-based-uncased was the best performance model:

1. distillbert-medical-fear-emotion: the model is available on HuggingFace repository under the name distillbert-based-uncased (details in the “Results” section). We call our fine-tuned model distillbert-medical-fear-emotion.

### Fear Level

Each sentence was classified into confidence score using the 2 emotion models (bhadresh-savani/distilbert-base-uncased-emotion and distillbert-medical-fear-emotion). The confidence score represents the probability that the model is confident that a given input belongs to a particular class, in our case, fear. The confidence score ranges from 0 to 1, the higher the score, the more confident it is that the input is related to fear. For example, a sentence classified with a score of 0.70 means that the likelihood that this sentence belongs to fear is 70%. From now on, we refer to the confidence score as the fear level. The fear level was determined using the 2 transformer models to determine consistency. For both models, we calculated the study independent variable, website level of fear.

We calculated the level of fear as follows:

1. Each website’s text content was analyzed at the sentence level.
2. For each sentence, a pretrained language model was used to estimate its probability of expressing fear. The confidence score ranges from 0 to 1, with higher values indicating a higher level of fear.
3. We calculated the level of fear for a particular website based on the mean of the fear confidence scores across all sentences on that website.
4. We categorized the websites into 2 categories: formal and informal.
5. The final level of fear variable represents the average of the fear levels calculated for all websites within each group (formal or informal).

The model of j-hartmann/emotion-english-distilroberta-base was used to examine the distribution of emotions (anger, disgust, joy, fear, sadness, surprise, and neutral) for formal and informal websites.

### Sentiment Analysis Using SiEBERT Model

For the purpose of sentiment analysis of each website content, we used the SiEBERT model [50]. SiEBERT model is a fine-tuned checkpoint of RoBERTa-large [17]. It is available on HuggingFace repository under the name siebert/sentiment-roberta-large-english. It provides reliable binary sentiment analysis for English language texts. It predicts either positive or negative sentiment for each instance.

### Negativity Level

In the same way, as we did with the emotion analysis models, SiEBERT classified each sentence according to its likelihood of being negative.

We calculated the negativity level as follows:

1. The text content of each website was analyzed at the sentence level.
2. A pretrained language model was used to estimate the probability of each sentence expressing negativity. There is a range of confidence scores from 0 to 1, with higher values indicating a greater level of negativity.
3. We calculated the level of negativity for a particular website based on the mean of the negativity confidence scores across all sentences on that website.
4. The websites were categorized into 2 categories: formal and informal.
5. The final level of negativity variable represents the average level of negativity calculated for all websites within each group (formal or informal).

### Statistical Analysis

Prior to calculating the averages of fear and negativity in each site type, we filtered repeated results to avoid duplication of results across different websites and content types.

We conducted a 2-tailed *t* test to evaluate the difference between the average fear detected on formal health-related websites and the average fear detected on informal health-related websites. For each result, *P*<.05 was considered statistically significant. SPSS (version 28; IBM Corp) software was used for statistical analyses.

## Results

### Description of the Created Database

After searching in Google Search all the selected symptoms, we received a database containing a total of 1448 unique websites. A total of 534 websites were classified as formal websites and a total of 914 websites were classified as informal websites. The data set has been cleaned by removing results containing unrelated content or errors caused by any restrictions from the websites. A total of 593 results were removed. In total, for the formal websites, there were 1820 pages containing 30,701 sentences, while for the informal websites, there were 1494 pages containing 25,961 sentences.

As a result of reducing duplicate results in which the same website returned with the same content in different search results, we were left with 1461 formal websites and 1294 informal websites.

### Words Representing Each Disease Family

The top 1000 significant words from each disease family retrieved from the content of the websites are shown in Multimedia Appendix 1. In order to determine the most significant words for each disease family, the average TF-IDF was calculated separately for each disease family. It can be seen that the most significant words for cancer are breast, cough, and pain. In addition, the most significant words in infectious diseases are breath, muscle, fever, and shortness. The most significant words in cardiovascular diseases are heart, chest, and pain.

### The Results of Bhadresh-Savani/Distilbert-Base-Uncased-Emotion Model for Classifying the Level of Fear

In the Methods section, the level of fear was defined. For this part, we used the pretrained model bhadresh-savani/distilbert-base-uncased-emotion. According to Multimedia Appendix 2, the fear level in formal websites is higher than that in informal websites based on this emotion detection model.

We used the *t* test to determine whether there was a significant difference between the fear level in formal and informal websites. The degree of freedom was 2753.

The results of the *t* test indicate a statistically significant difference between formal and informal websites in terms of the fear level in this model (Table 2 presents the *t* test table).

**Table 2:** The *t* test results of the comparison between formal and informal websites in terms of fear and negativity level.

The model			Groups mean				Groups SDs			*t* test values (*df*)		*P* value
**Fear level**												
	bhadresh-savani/distilbert-base-uncased-emotion	0.388		0.366		0.177		0.168	3.331 (2753)		.001	
	distillbert-medical-fear-emotion	0.584		0.569		0.121		0.134	3.040 (2753)		.002	
**Negativity level**												
	SiEBERT	0.657		0.636		0.211		0.201	2.726 (2753)		.006	

### The Results of Distillbert-Medical-Fear-Emotion Model for Classifying the Level of Fear

The emotion data set from an web-based health community was used to train several transformer models [29]. As a result of the data set, we can train existing models that estimate fear levels arising from health-related information and find the best model based on accuracy and *F*_1_-scores. Eighty percent of the data set was used for training, 10% for validation, and 10% for testing.

The distillbert-based-uncased model provided the highest performance with accuracy on the validation set of 0.944 and an *F*_1_-score of 0.943. On the testing set, the model achieved an accuracy of 74.4%. There was 75% intercoder agreement on the same task with human annotation in the study by Sosea and Caragea [29]. We call our fine-tuned model distillbert-medical-fear-emotion. Table 3 shows the accuracy and *F*_1_-scores of transformer models on the testing set.

**Table 3:** Transformer model accuracy and F1-scores on the test set.

Models	Description	Accuracy	*F*_1_-scores
distilbert-base-uncased	Distilbert is a distilled smaller and faster version [18] of the BERT base model.	0.744	0.771
ClinicalBERT	Bio_ClinicalBERT is a model trained on data from the Beth Israel Hospital’s ICU patients [51].	0.729	0.762
bert-base-uncased	Pretrained model on English language using a masked language modeling objective [15].	0.736	0.757
roberta-large	RoBERTa is a transformers model pretrained on a large corpus of English data in a self-supervised fashion [17].	0.736	0.765
albert-base-v2	ALBERT is a transformers model pretrained on a large corpus of English data in a self-supervised fashion [19].	0.738	0.757

Our best model, distillbert-medical-fear-emotion, was then used to automatically classify each sentence by its fear level. For both formal and informal websites, fear levels are presented. Multimedia Appendix 3 shows the model result. It can be seen that formal websites have a higher fear level.

Another *t* test was performed and revealed a statistically significant difference between formal and informal websites in terms of the fear level of distillbert-medical-fear-emotion (Table 2 presents the *t* test table).

According to the results, both models (the bhadresh-savani/distilbert-base-uncased-emotion model and the distillbert-medical-fear-emotion model) were consistent in their results when comparing formal and informal websites.

### The Results of the SiEBERT for Classifying the Level of Negativity

In the Methods section, the level of negativity was defined. Using the SiEBERT model, we analyzed the level of negative sentiment arising from formal and informal websites. Multimedia Appendix 4 shows that informal websites also exhibit a higher degree of negativity in the sentiment analysis. We used the *t* test to determine whether there was a significant difference between formal and informal websites. Our results indicate that there is a statistically significant difference between formal and informal websites in terms of negative sentiment reflected in the text (Table 2 presents the *t* test table).

**Figure 2:**
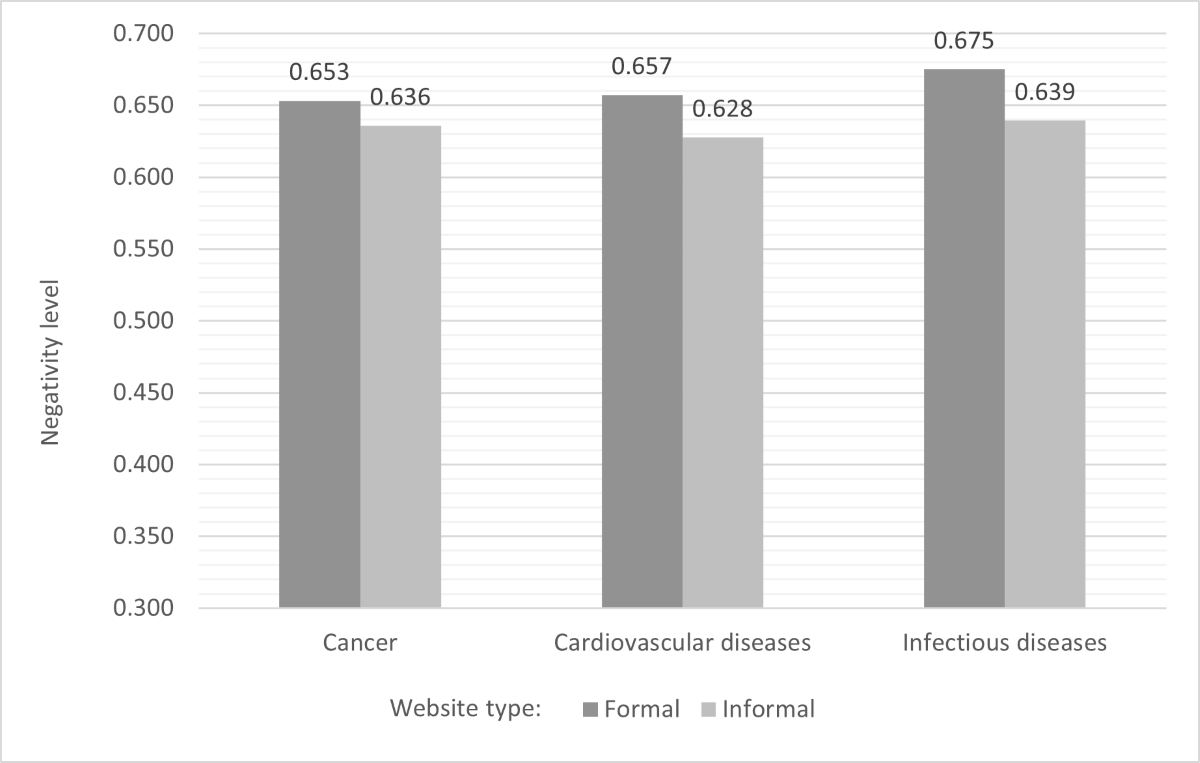
Negativity level by disease family.

Figure 2 presents the negativity level by disease family, including cancer, cardiovascular diseases, and infectious diseases. It can be observed that the negativity level in texts related to infectious diseases is higher than that in texts related to cancer and cardiovascular diseases.

### The Results of the J-Hartmann/Emotion-English-Distilroberta-Base for Emotion Detection

As part of the emotion analysis process, we ran the j-hartmann/emotion-english-distilroberta-base emotion analysis model across the website texts to gain a deeper understanding of the emotions expressed in these texts.

**Figure 3:**
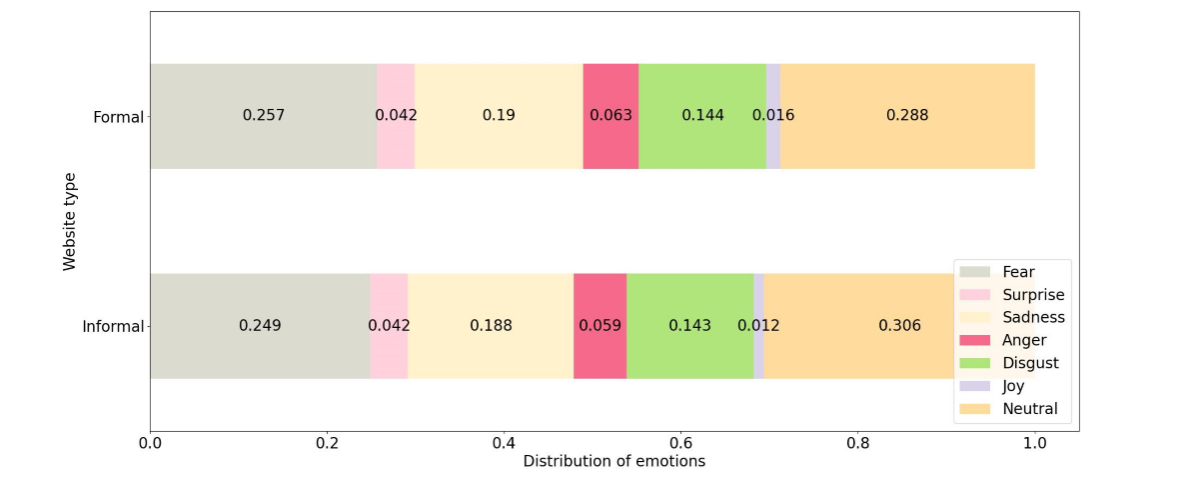
Emotions (fear, surprise, sadness, anger, disgust, joy, and neutral) associated with formal and informal websites.

In Figure 3, we show the distribution of emotions across formal and informal websites. As can be seen in both cases, neutral emotion dominates, followed by fear, sadness, and disgust, indicating that both types of websites convey mostly negative emotions. A comparison between formal and informal websites reveals that fear is higher in formal websites, as was shown in previous models, but anger and sadness are also higher in formal websites.

## Discussion

### Principal Findings

As part of our results, we observed that websites related to infectious diseases induced a higher level of negativity than websites related to cancer.

A possible explanation for this finding is that cancer is widely perceived by the public as a severe disease, and, as such, the public is more conformed with formal guidelines and recommendations regarding cancer prevention and treatment, and no fearful tactics and messages are required to achieve compliance. On contrary, the severity of infection diseases is underestimated by large parts of the public. Moreover, antivaxxers spread misinformation regarding various vaccines that are crucial to primary prevention of infectious diseases, including influenza, COVID-19, measles, smallpox, and more, and may prevent severe outcomes and mortality.

As a result, health professionals may include frightening text related to infectious diseases, in order to increase the public compliance with formal recommendations. Moreover, the recently emerging outbreaks of some infectious diseases may have been resulted in a more stressful and fear-inducing tone in formal messages.

Efforts may be made to obtain a balance between communicating diseases’ severity and prevention measures and avoiding excessive scare tactics. This may be achieved by combining relevant facts with appropriate emotional appeals. Such a balance may increase the public awareness and compliance.

Furthermore, our results demonstrate that formal websites introducing health-related information induce more negative sentiments and fear emotions than informal websites.

A possible explanation for the higher negative sentiments and fear emotions found on formal websites could be that formal websites are more precise and provide more correct information on health-related issues [52]. As such, they may contain more clinical and scientific terminology regarding the searched medical issue than informal websites. Such details may include disease symptoms, the diagnosis process, prognosis, side effects, risks, possible treatments, and worst-case scenarios. These details may increase the negativity and fear levels induced by their provided text [52]. A formal website may also be associated with higher levels of negative sentiment and fear emotions since it is managed by government and health authorities, which may use fear appeals to motivate behavior changes such as vaccinations. These tactics may increase anxiety if they are not carefully framed [53].

On the other hand, informal websites are less informative and may contain inaccurate information and misinformation about health-related issues. For example, the claim that consuming apricot seeds will cure cancer is a misinformative non–evidence-based claim that can be found in some informal websites [54]. As such, informal websites may have decreased negativity and fear level in their provided texts, and, by that, they may appear more relaxing to the reader.

The public seeking for health-related information uses formal as well as informal websites. Formal health-related websites contain accurate, evidence-based information regarding different diseases, and it is desirable that the public will use formal health-related information [52,54]. On the other hand, informal health-related websites may include misinformation and may lead the public to erroneous decisions [54]. For example, misinformation regarding the measles, mumps, and rubella vaccine led to some severe measles outbreaks in some European countries and the United States [54]. However, the public tends to choose information sources that are not stressful and introduces the information in a manner that does not increase the reader’s stress [1,55,56]. Therefore, the public may prefer to retrieve information from informal health-related websites, which, according to our results, induces less stress, regardless of misinformation which may exist.

This may lead to undesirable public health consequences. Selective focus on a specific type of medical content that is less stressful or anxiety-inducing can result in biased search strategies when retrieving health information [57]. Biased health information retrieval strategies can negatively affect public health and decision-making, resulting in inaccurate, incomplete, or misleading information [58,59].

Confirmation bias is a common bias when users search for information online. A confirmation bias is defined as the unconscious inclination to favor information that supports one’s own viewpoint or belief [60]. As a result of confirmation bias, users are more likely to accept misleading information since it is consistent with their previous preconceptions [53]. Confirmation bias can be associated with the tendency to prefer positive information, in which users do not only seek information that confirms their existing beliefs but also seek to avoid negative information in order to maintain a positive emotional state.

In light of our recommendation to reduce levels of negativity and fear in the texts, it is important to balance fears raised by the text, while also providing accurate and comprehensible information to reduce the risk of biased information.

It is essential that health information is provided in a comprehensive manner [53], and some topics require a more serious tone of voice. For example, a serious tone may be necessary when discussing the full risks associated with a health condition, but a positive tone may be more effective in encouraging preventive health behaviors, such as vaccines. Therefore, the strategy for delivering information should include accurate, complete, and comprehensible information, as well as a focus on enhancing the positivity of the information.

The COVID-19 pandemic demonstrated that transmitting clear, precise, and evidence-based information to be used by the general public may be used as a strategy for controlling the disease, and it is one of the challenges of formal health organizations [55]. During the COVID-19 pandemic, an abundance of contradicting information existed, including misinformation regarding the disease severity and the required measures, including the vaccines that were developed during the outbreak. A study that examined the differences between formal and informal websites with regard to COVID-19 prevention measures found significant differences regarding some essential measures such as wearing masks [52]. Another study found an association between the source of information regarding the COVID-19 vaccines (formal or informal) and the willingness to receive the vaccine [56]. Hence, one of the challenges of public health professionals while facing the COVID-19 pandemic was to communicate challenging messages that build public trust [61]. Moreover, the COVID-19 pandemic was considered a unique and exceptional event worldwide [62]. As such, it created a new normal that we need to learn to live with [62]. One of the implications of living with the new normal is understanding that public health exists within a social context. Embracing public health as a social concept is essential for dealing with COVID-19, as well as with other global crises, such as climate change [62]. Therefore, a challenge for living with the new normal is achieving solidarity in health and public health and, by that, confronting cross-national and cross-cultural risks [62]. The use of positive texts by formal health-related websites, which can be achieved by reducing the negativity and fear levels in the texts, may increase the credibility of formal health websites, perceived by the public, and increase their usage by the general public, as well as the public’s trust, engagement, and compliance with the measures during the pandemic. This may contribute to the solidarity, which is essential to living with the new normal.

Encouraging public professionals and public health organizations to use NLP tools before publishing health-related information, in order to identify negativity and fear levels in the text and reduce them, may be helpful to achieve a more positive and less stressful text to be transmitted to the public and may increase the public compliance to suggested measures.

### Limitations

This study has some limitations. Using NLP tools as transformer models for estimating negativity and fear in the text comes with certain limitations. While these models have revolutionized the field of NLP and have accomplished remarkable feats in various tasks, they still have difficulty to capture the nuances of negative or fearful sentiments in the same manner as a human being. In addition, fear is a subjective feeling and may vary between people. Nevertheless, we do believe that our algorithm catches texts that appear negative and fearful to the majority of the population, since it is based on words that are likely to be perceived as negative and fearful by a large portion of the population, such as death, lethal, complications, and more. Further research is required to examine the correlation between negativity and fear levels received by our algorithm and negativity and fear levels observed by human participants.

Another limitation is that this study focused on 3 specific diseases: cancer, cardiovascular, and infectious diseases. We focused on these diseases since they are leading causes of death and as such, people are likely to search on the web for information about their symptoms [8]. We believe that focusing on these diseases provided a reasonable starting point for this initial exploratory study. Further research is required to expand the scope of this research by examining other diseases.

Furthermore, in this study, we examined the emotions and sentiments expressed in the text; however, people may have additional priorities or preferences when consuming web-based health information. Health information consumption should consider a broad range of user needs and preferences, such as how clear, easy to understand, and complete the information is. The differences between formal and informal health-related websites should be further studied to gain a deeper understanding of the diverse needs and priorities of health information users when accessing health information, for example, to examine the differences between the clarity of the information on the 2 types of websites.

### Conclusions

We believe that improving the positivity of messages provided by formal health-related websites by reducing the negativity and fear levels may increase the public use of these websites, as well as the public’s trust in them, and the public’s compliance with recommended measures. It can be achieved by applying NLP tools to texts published by official public health websites. Public health decision makers may use existing NLP models, which include appropriate graphical user interface to assess the negativity and fear levels in a text, before publishing it to the public. An example of such a model can be found in SiEBERT—English-Language Sentiment Classification [63]. However, training public health professionals to use such tools and to reduce the negativity and fear levels in their published texts is essential to achieving this goal.

## Appendix

Multimedia Appendix 1 Top 1000 words for each disease.

Multimedia Appendix 2 Fear level in formal and informal websites based on distilbert-base-uncased-emotion.

Multimedia Appendix 3 Fear level in formal and informal websites based on distilbert-medical-fear-emotion.

Multimedia Appendix 4 Negativity level in formal and informal websites.

## Abbreviations

NLP: natural language processing
TF-IDF: term frequency—inverse document frequency

## Data Availability

The data sets generated and analyzed during this study are not publicly available because they contain sensitive information that could compromise the privacy and consent of the websites. The transformed data are, however, available upon reasonable request from the authors.
