# Supplementary figures and images for: Differences in Fear and Negativity Levels Between Formal and Informal Health-Related Websites: Analysis of Sentiments and Emotions
Source: J Med Internet Res. 2024 Aug 9;26:e55151. doi: 10.2196/55151 (PMC11344190; doi:10.2196/55151)

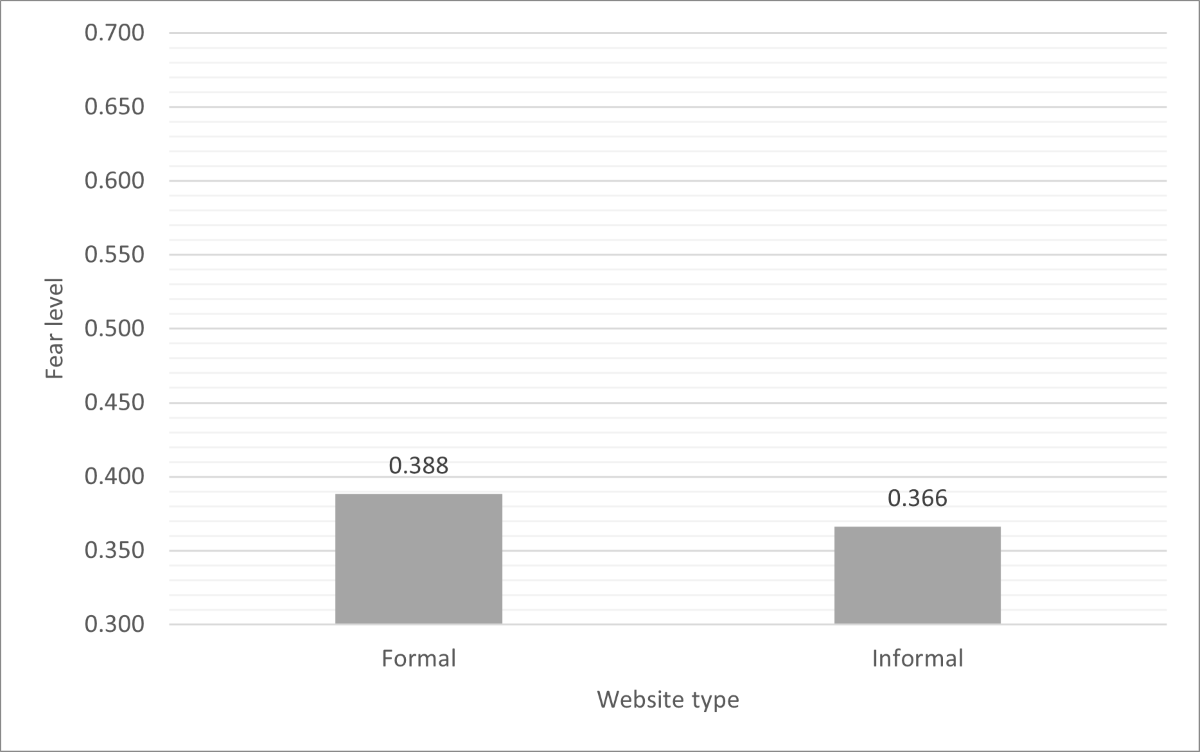

Supplement: Multimedia Appendix 2 [file jmir_v26i1e55151_app2.png]

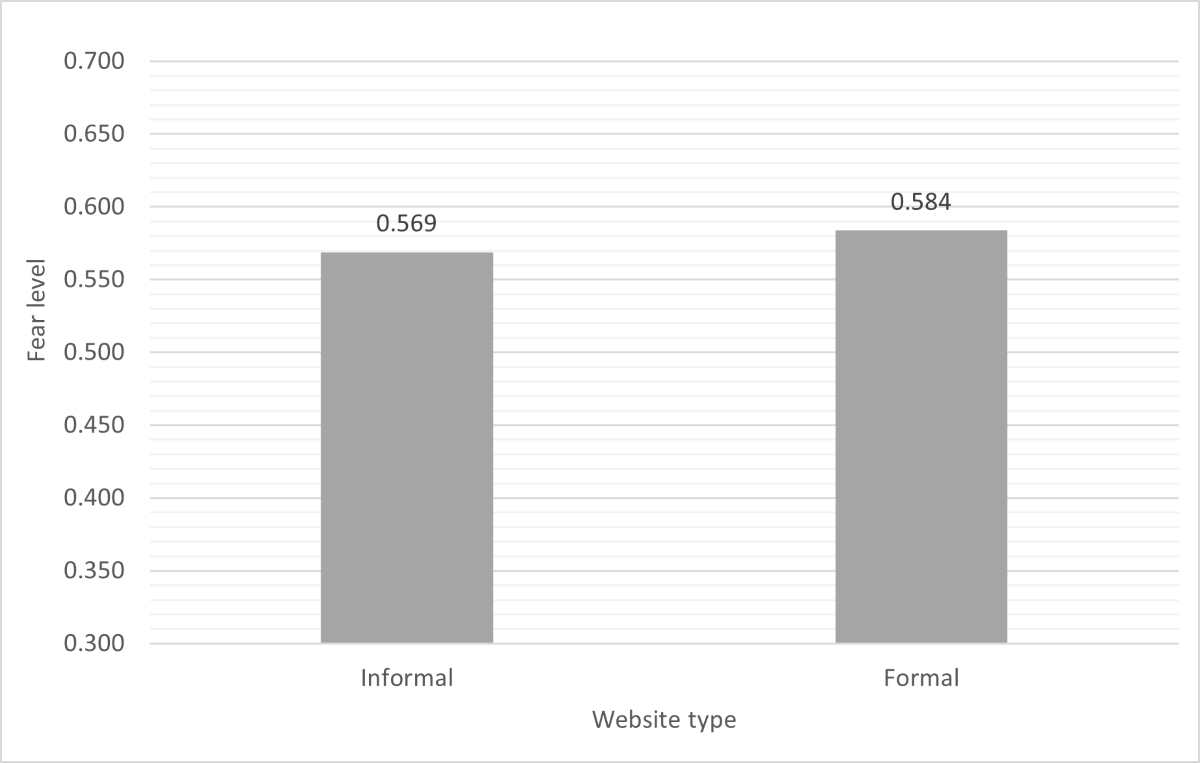

Supplement: Multimedia Appendix 3 [file jmir_v26i1e55151_app3.png]

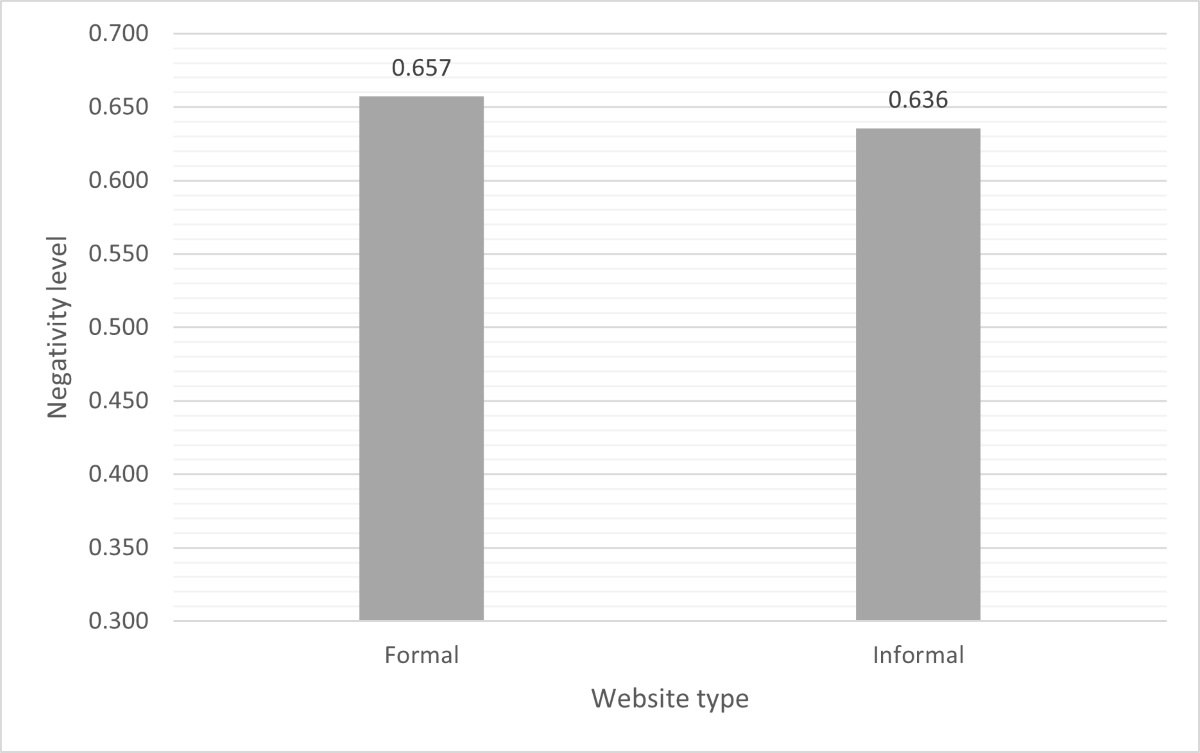

Supplement: Multimedia Appendix 4 [file jmir_v26i1e55151_app4.png]
